# Efficiency of complex production in changing environment

**DOI:** 10.1186/1752-0509-3-3

**Published:** 2009-01-07

**Authors:** Shai Carmi, Erez Y Levanon, Eli Eisenberg

**Affiliations:** 1 Minerva Center & Department of Physics, Bar-Ilan University, Ramat Gan, Israel; 2 Center for Polymer Studies, Boston University, Boston, MA, USA; 3 Department of Genetics, Harvard Medical School, Boston, MA, USA; 4 Raymond and Beverly Sackler School of Physics and Astronomy, Tel-Aviv University, Tel-Aviv, Israel

## Abstract

**Background:**

Cell function necessitates the assemblage of proteins into complexes, a process which requires further regulation on top of the fairly understood mechanisms used to control the transcription and translation of a single protein. However, not much is known about how protein levels are controlled to realize that regulation.

**Results:**

We integrated data on the composition of yeast protein complexes and the dynamics of their protein building-blocks concentrations to show how the cell regulates protein levels to optimize complex formation. We find that proteins which are subunits of the same complex tend to have similar levels which change similarly following a change in growth conditions, and that abundant proteins undergo larger decrease in their copy number when grown in minimal media. We also study the fluctuations in protein levels and find them to be significantly smaller in large complexes, and in the least abundant subunit of each complex. We use a mathematical model of complex synthesis to explain how all these observations increase the efficiency of complex synthesis, in terms of better utilization of the available molecules and better resilience to stochastic variations.

**Conclusion:**

In conclusion, these results indicate an intricate regulation at all levels of protein production for the purpose of optimizing complex formation.

## Background

Much of the cell's activity is directed towards synthesis of single proteins. Yet, the functional units of various cellular processes are not single proteins but rather protein complexes, i.e. a couple or more proteins bound together, performing specific tasks[1]. While much is known about the dynamics of gene expression and protein synthesis, the understanding of the mechanisms of complex formation and their dynamics is still in its early stages. Given the key role played by complexes in all eukaryotes, the investigation of cellular complexes is of major importance. Indeed, in recent years, there has been a significant experimental progress in identification of protein-protein interactions[2,3] and their roles, as well as in detecting full functional complexes [4-7]. Statistical analysis of the resulting protein networks and complexes has attracted much interest[8]. In particular, data describing protein interactions and complexes was integrated with several other sources of information such as gene expression [9-14], protein function[6], localization[15,16], essentiality[16,17], and others[18] to offer insight into the characteristics of the complexes.

A major obstacle to these analyses stems from the fact that most studies of gene product abundance measure mRNA abundance, which is insufficiently correlated with actual protein levels. The widely used DNA microarray experiments, providing a transcriptome-wide measure of mRNA levels, are limited by the need to extrapolate from mRNA levels to protein and complex levels, bridging over post-transcriptional effects, which are, in large, still not sufficiently understood. Key questions regarding the dynamical behavior of complexes, such as what are the advantages of a given complex structure and how it changes under different conditions, need to incorporate direct data on protein levels.

In a previous work[19], we have shown that protein complexes in yeast[20] tend to have similar protein abundances[21]. That is, the variance of the protein levels among subunits of a complex is significantly lower than expected by chance based on the distribution of levels of single proteins. Analyzing a simple mathematical model of complex formation, we have explained this tendency as a selection towards efficiency of complex production. Intuitively, excess in the level of one component of a given complex results in a surplus of undesired sub-complexes that contain this overly-expressed protein, followed by a shortage of other sub-complexes required for the synthesis of the complete goal complex. As a result, the efficiency of complex synthesis (defined as the ratio of the number of whole complexes to the amount of the component with lowest abundance; see Methods) decreases [22,23].

In this work, we present more evidence for cells' internal optimization for efficient complex production. We use newly available measurements providing results for the comparison of yeast protein levels in different environmental conditions, as well as measures of the cell-to-cell variation in actual protein levels[24]. The availability of these proteomic data enables us to directly and globally investigate the cells' response to external stimuli at the proteomic level. We integrate other sources of information on protein length[25], stability[26] and translational activity[27], and apply a mathematical model of complex formation, to further reinforce the general optimization principle.

We find that yeast complexes tend to contain proteins with similar change (increasing or decreasing their levels) when grown in minimal media. Also, down regulation of proteins in minimal media is stronger for proteins that are more abundant in rich media, which we explain as tendency towards efficiency using a mathematical model of complex formation. Using our model we find that complex levels are most sensitive to fluctuations in the levels of the least abundant protein, and indeed, experimental data show significantly smaller noise in the levels of the least abundant protein in a complex. Similarly, variation in protein production is significantly less for proteins in large complexes. Finally, we show that the length and lifetime of complex subunits are more similar than expected by chance. Taken together, these results attest for highly efficient control over the concentration of complexes through tight regulation of the expression of their building blocks.

## Results

### Uniform direction of change in levels of complex subunits

In an experiment reported by Newman et. al. [24], the abundance of yeast proteins was compared between two states: YEPD (rich), and SD (minimal) by measuring cells' fluorescence due to GFP-tagging of individual proteins. Based on these measurements, Newman et. al. [24] assigned each protein to one of three classes: *Rich-State *proteins (proteins whose concentration in SD condition is significantly lower than the one in YEPD), *Minimal-State *proteins (YEPD concentrations are significantly lower than those in SD), and *Constant *proteins (no significant difference in concentrations observed). Out of about 6,000 yeast proteins, more than 2,000 were classified. 232 proteins are minimal-state, 684 are rich-state, and 1298 are constant. Integration of this data on protein levels with the collection of protein complexes (downloaded from MIPS[28] website, [see Additional file 1]) generates interesting observations.

In the following, we present a number of results relating abundance and noise levels of complex subunits. All of these results are then analyzed in terms of a simple toy-model of complex formation, and are shown to be different manifestations of complex production efficiency.

**Figure 1:**
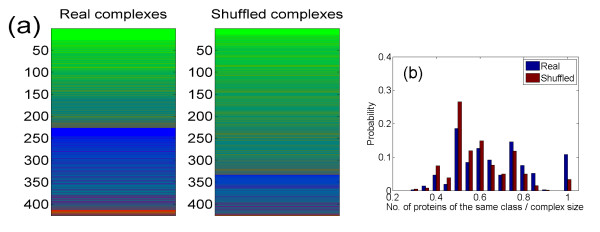
**(Color) Protein make-up of the yeast complexes**. (a) Each horizontal line represents one complex, and its color is determined by the RGB scheme, where the green component of the line color is the fraction of complex subunits which are constant proteins, the blue is the fraction of rich-state proteins, and the red is the fraction of minimal state proteins. For real complexes (left panel), many complexes show almost pure base colors, corresponding to complexes in which all (or most) of the proteins belong to the same class. Following shuffling of the protein classes (right panel), colors tend to be mixed, indicating mixture of proteins of different classes. (b) Uniformity in change of protein levels in complexes between YEPD and SD states. For each complex, we calculated the largest of the three fractions of its subunits that exhibited increase, decrease or no change upon a change between YEPD and SD states. In the figure, we plot the distribution of this fraction among all complexes for real complexes and after shuffling of the protein complexes (averaged over 100 randomizations). For real complexes, typically a large fraction of the subunits change uniformly, in contrast to the situation for the shuffled ones (p-value ≈ 10^-11^, Kolmogorov-Smirnoff test).

We first extract the number of complexes with uniform change, i.e., complexes in which all identified proteins belong to the same class. Excluding complexes with three or less identified subunits, 426 complexes are available for analysis (complexes with only few subunits might exhibit uniform change just by chance, masking any real trend. We have verified that our results remain qualitatively the same even when analyzing all complexes). There are 46 uniform change complexes, compared to 14 ± 4 complexes (P-value ≈ 10^-15^, see Methods) expected when randomly assigning proteins to classes – see Figure 1(a) for a graphical comparison of real and random complex make up [see Additional file 2]. In 26 of these complexes all proteins are constant, in 19 all are rich-state, and in one all are minimal-state (ubiquinol-cytochrome c reductase complex (bc1 complex), which is a component of the mitochondrial inner membrane electron transport chain[29]). Looking at the whole set of complexes, one also observes a clear tendency towards uniformity in direction of change (Figure 1(b)).

### Larger decrease in the levels of more abundant proteins

**Figure 2:**
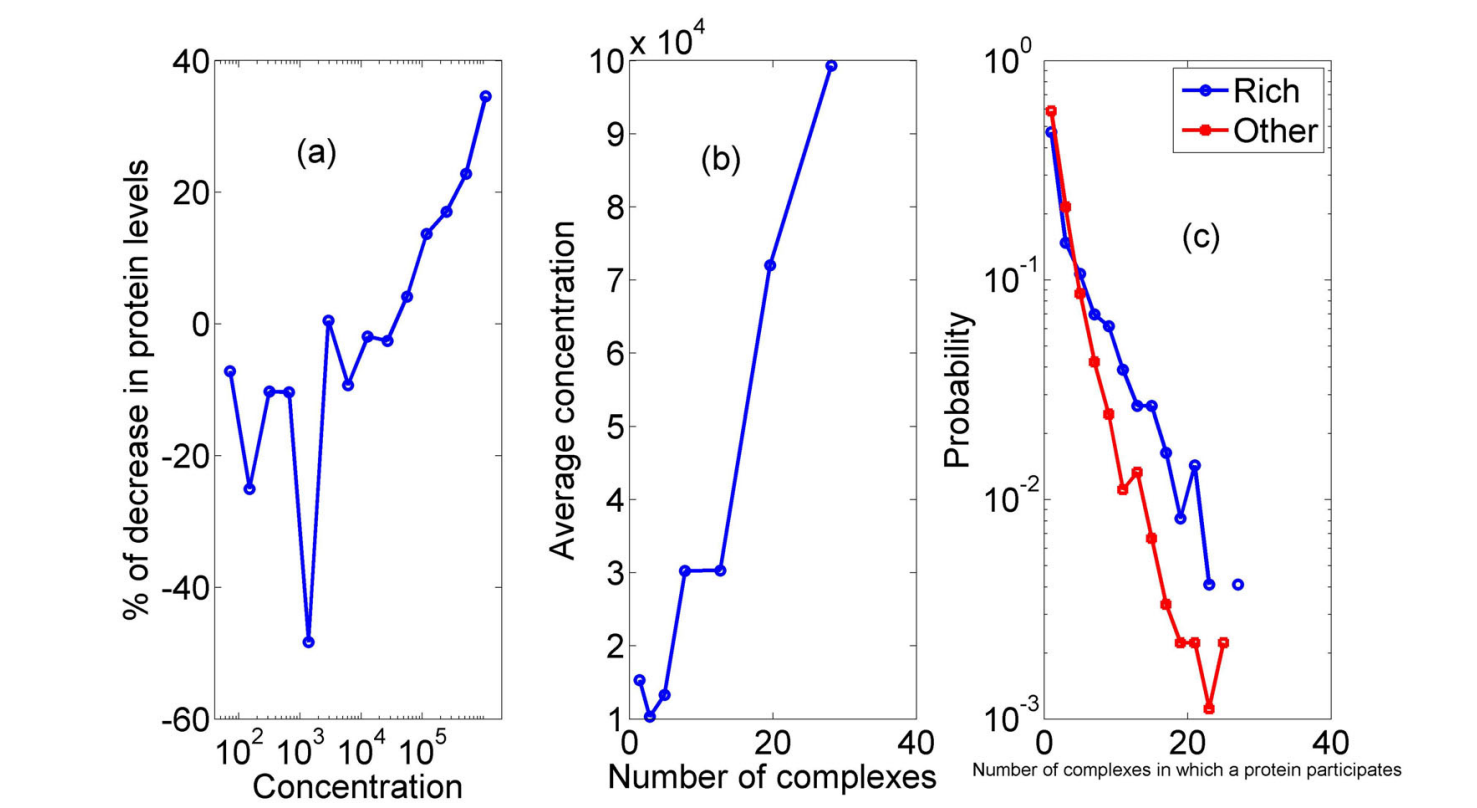
**Decrease in levels in SD is larger for abundant proteins**. (a) Percentage of decrease in the protein level in minimal state ((*level*_*yepd level*_*sd*)/*level*_*yepd *100%), compared to protein concentration (in units of no. of molecules per cell). Negative percentage represents increase in levels. (b) Average protein concentration (in units of no. of molecules per cell) vs. the number of complexes in which a protein participates (c) Probability distribution of the number of complexes in which a protein participates for rich-state proteins and for constant + minimal-state proteins.

The actual amount by which protein levels change is also of interest. Looking at the difference in protein levels between the SD state and the YEPD state as a function of the steady-state concentration in YEPD state[21] (Figure 2(a)), one clearly sees that the higher the protein levels are, the larger is the decrease in the minimal state, where increase is observed only for those proteins which are scarcely expressed in YEPD. For proteins with higher levels in YEPD, the correlation coefficient between the logarithm of the concentration and (*level*_*yepd level*_*sd*)/*level*_*yepd *is r = 0.1, P ≈ 10^-4^.

### Lower noise in components of large complexes

Next, we study role of 'noise', that is, the cell-to-cell variation in protein levels, and its interplay with protein complexes production. Measurements of cell-to-cell variance in protein levels were also reported by Newman et. al. [24]. Noise is measured by the CV (coefficient of variation), the standard deviation of protein levels divided by the average, in percentage. In the absence of correlations between different individual proteins, one would expect the standard deviation to be proportional to the square root of the mean, or *CV *∝ *N*^-1/2 ^× 100%, where N is the average number of copies in a cell. In reality, it was shown[24] that the CV decreases with protein level, but much slower than *N*^*-1/2*^, reflecting the fact that production of multiple copies of a single protein is typically a correlated process.

We first note that proteins participating in a complex have significantly lower noise[30] as compared to other proteins (CV 19.7 vs. 21.5 for complex and non-complex proteins, respectively (in YEPD state). P < 10^-14^, t-test; [see Additional file 2]). Among the complex proteins, we find that components of large complexes (144 complexes with at least 15 subunits) exhibit a significantly lower noise (CV 18.52 vs. 19.07 ± 0.12, P ≈ 10^-5^). We note that the large complexes contain proteins of higher concentration compared to what is expected by chance (averaged log-concentration 8.7 vs. 8.57 ± 0.03, P ≈ 10^-6^), which can partly explain the lowered CV. However, the noise in components of large complexes is even lower than could be expected based on abundance alone (P ≈ 10^-4^, see Methods). This tendency holds even upon controlling for the large amount of essential proteins found in large complexes (P ≈ 0.003, see Methods [see Additional file 2]).

### Lower noise in the least abundant protein in a complex

We then return to all complexes, and focus on the level of the least abundant protein in each complex. First we note that the concentration of these proteins (averaged over all complexes) is 36% higher (1,310 molecules/cell, averaged over all complexes with at least 4 identified subunits) than could be expected by chance (960 molecules/cell, P ≈ 10^-8^; see Methods), or due to the complexes having more similar subunit abundances than random [see Additional file 2]. As a result, the CV of the protein with minimal concentration, which is an indication of the typical possible loss (in percentage) of complexes due to noise in protein synthesis, is lower (20.7%, in comparison to 21.3% ± 0.2%, P ≈ 0.004). In addition, this remains true even when abundance and essentiality are controlled for (P ≈ 0.02, see Methods). In comparison, the protein of highest concentration in a complex does not have CV lower than expected by chance (in fact, it has (non-significantly) higher CV).

Thus, we conclude that for large complexes, as well as for the least abundant protein in each complex, significantly low noise levels are found. In both cases, it is also found that the low noise level is not only due to proteins having high abundance or being more essential than expected by chance.

### Complex subunits have similar length

Another aspect of complex organization is revealed by studying protein and mRNA lengths. Recently, the entire yeast transcriptome has been sequenced[31], from which we extracted the full length of the mRNA molecules, UTRs included. We found that the lengths of transcripts that belong to the same complex tend to be more similar than expected by chance. To show this, we calculated the variance of the (logarithm of the) lengths (5' untranslated region length + open reading frame length + 3' untranslated region length) of genes that are subunits in a given complex. We consider again only those complexes with information on at least 4 subunits (there are 467 such complexes). The variance for real complexes is 0.27 (averaged), significantly lower than the randomly expected one (0.33 ± 0.013, P ≈ 10^-7^). We note that the results holds even if only the UTRs are considered, but the significance is much lower (P ≈ 0.05).

A negative correlation exist between protein abundance and length (see e.g. [32]). Also, proteins of same function tend to have similar length[33]. Thus, one may argue that the similarity between the transcript lengths of complex subunits is due to either their similar abundance or similar function. Therefore, we controlled for both abundance and function when shuffling the complexes [see Additional file 2]. The variance in the complexes randomized with control of abundance and function is indeed somewhat lower than for completely random shuffling (0.31 ± 0.012 vs. 0.33 ± 0.013), but is still significantly higher than that observed in real complexes (0.27, P ≈ 10^-5^).

### Balance of protein translation activity and degradation rate

The concentration of a protein results from a balance between its rate of synthesis (translational activity[27], which, in turn, relates to its mRNA abundance and ribosome occupancy and density) and rate of degradation[26]. To identify the regulation strategies employed to achieve the uniformity in protein levels in a complex, it is interesting to study the relative contribution of each of these two factors.

According to the simplest model for the kinetics of protein synthesis[27], the logarithm of a protein's concentration is the sum of the logarithms of its translational activity and the its half life. Thus, the variance due to each of them can be computed independently. We find that both show lower variance in complexes than expected by chance. However, while the variance of the translational activity is 34% lower than expected (0.47 vs. 0.72 ± 0.03, P < 10^-14^), the variance of the half life is only 10% lower than expected, and less significant (1.24 vs. 1.38 ± 0.08, P = 0.05). Thus, it seems that the half life plays a less significant role in the regulation for efficient complex synthesis.

## Discussion

We have thus far analyzed several experimental sources integrating protein complexes with data on protein abundance, noise, response to environmental change, and others. As we explain below, these observations may be interpreted as signatures of optimization of complex formation at the global level of the yeast cell. A simple mathematical model of complex formation is employed to support some of our claims.

### Change of protein levels in a complex across different conditions

Our first finding was that protein abundances in a complex are correlated between two different environmental conditions. A correlation between expression patterns across different conditions was previously shown at the mRNA level [10-12]. Here we presented first evidence supporting this correlation at the protein level. As was shown in Ref. [19], complex subunits levels tend to be similar in the rich YEPD medium so that the complex production is efficient. In order to maintain this level of efficiency in face of the different environment, the direction of change in protein levels from YEPD to SD should also be similar across complex subunits.

Next, we found that proteins of larger abundance exhibit steeper decrease in SD condition. This is also a manifestation of efficiency, as we now demonstrate in terms of a simple toy-model of complex formation (see Methods for model details). A complex in our model is made up of 3 different components: A, B, and C, with total concentrations A_0_, B_0_, and C_0_, respectively. [ABC] is the concentration of the complete complex ABC, the desired outcome of the production. We analyze the case where one is given a set of initial subunit concentrations A_0_, B_0_, and C_0_, and wants to change them to achieve a certain desired decrease in the amount of the target complex.

**Figure 3:**
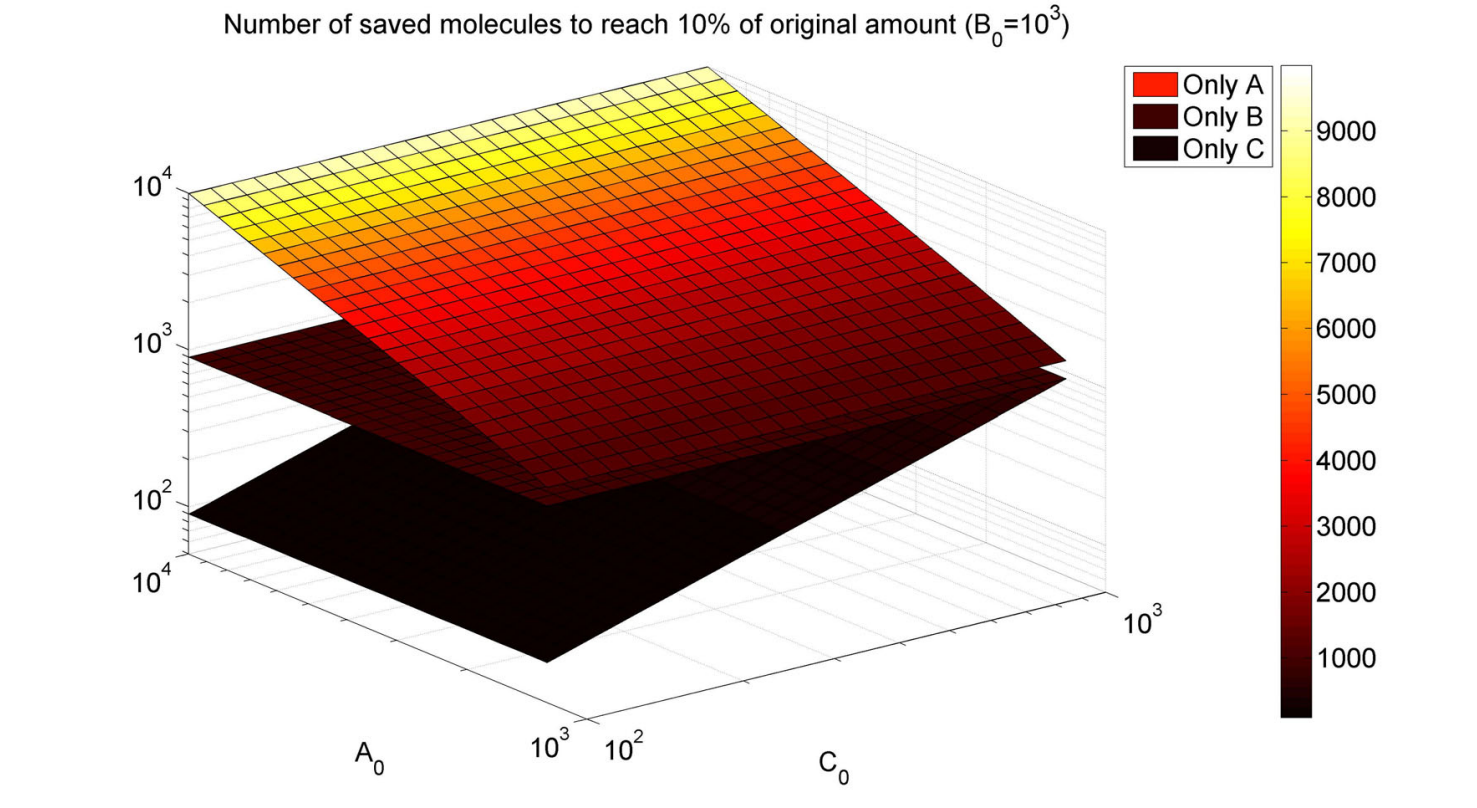
**(Color) Decrease in protein levels in minimal state – model results**. For various values of A_0 _and C_0 _in our complex formation model (B_0 _= 1000 fixed, and C_0_<B_0_<A_0_), we plot the amount of molecules (per unit volume) saved if we change the concentration of only one component (top to bottom manifolds: A, B, C, respectively), to decrease the concentration of the goal complex ABC by 90%. Decreasing the amount of the most abundant molecule (A) yields the largest gain.

For simplicity, we assume first that only one component is allowed to be changed. One may then ask which is the component whose levels should be reduced in order to reach the target concentration of the goal complex while using as few molecules as possible? Intuitively, one expects this to be the most abundant component. This is clearly the case when concentrations are very small, [ABC] ≈ A_0_B_0_C_0_, and thus the most economic way (in terms of the total number of A, B, and C molecules) to lower the product ABC is by decreasing the level of the largest of A_0_, B_0_, and C_0_. Indeed, this picture holds also when concentrations are not small. In Figure 3, we plot the number of molecules saved (per unit volume) when the final concentration of ABC is 10% of the original, for various initial values of A_0_, B_0_, and C_0_. Without loss of generality, we look at C_0_<B_0_<A_0_. The solution of the model equations results in 3 surfaces, corresponding to changing the amounts of A only, B only, and C only. It can be seen that it is always most economic to change A_0 _(the most abundant component).

The same picture holds in the general case, where the concentration of all three types is free to change. Optimal efficiency (in terms of saved molecules) is achieved when the concentration of the most abundant type decreases most [see Additional file 2]. Therefore, the observed correlation between abundance and difference in levels between the environments can be also attributed to selection towards efficiency.

Parenthetically, we note that within the above simplistic model, one would expect all subunits of a complex to have equal levels in order to optimize the production rate. In practice, although protein abundances in a complex are significantly more similar than expected by chance, they do not have exactly the same levels. Obviously, this can be partly attributed to the simplifying assumption of symmetric rate constants used in our model (see Methods), which, in reality, does not hold of course. As a result, the optimal point in not necessarily at all components having equal concentrations. More importantly, while protein levels are regulated to increase efficiency, they are certainly not exactly at the optimal point.

In fact, the above analysis assumes that the cell is off-optimum for the YEPD condition, and is getting closer to the optimal balance when in the minimal SD condition. Indeed, the variance in protein levels in a complex, averaged over all complexes is 5% lower in SD state compared to YEPD state (and significantly lower than expected by chance, P ≈ 10^-7^), supporting this view. Why would protein levels in SD state be more regulated compared to YEPD state? A possible reason is that proteins which participate in more than one complex or in a combinatorial sub-module of a complex[34] have competing constraints which prevent them from being at the optimum level desired for each single complex they participate in. It is reasonable to assume that in a minimal (SD) condition the constraints on protein levels due to participation in many complexes are partly released. In addition, the production of some of the non-essential complexes is terminated or reduced. Thus, protein levels can be closer to the optimum needed for each complex production separately.

In order to support this, we point out (Figure 2(b)) that proteins which participate in more than one complex generally appear in more copies than others. Furthermore, Figure 2(c) presents the probability distribution of protein's 'degree', i.e., the number of complexes in which a protein participates, for rich-state proteins and all other proteins separately. It can be seen that for rich-state proteins the degree distribution is wider (P < 10^-9^, KS test) with a larger average (5 complexes on average for rich-state proteins vs. just 3.2 for the rest). Clearly, most of the change in expression profile occurs in proteins which participate in many complexes, as expected by the above picture. In the minimal condition, these protein levels can be closer to the optimum needed for each single complex production. This partly explains why cells "choose" to be closer to optimality in the SD state, and provides another explanation to the correlation between relative change and absolute concentration.

### Noise in protein levels in a complex

Next we studied the noise levels of complex proteins. We demonstrated that large complexes usually contain proteins with lower noise. This finding attests for another level of efficiency in complex production. Since large differences in the abundances of the different constituents of a complex result in decreasing efficiency of its production[19], it is of high importance to keep the levels of the complex components stable. For large complexes, where each of the many components deviating from its desired concentration could result in a loss of many proteins, regulation of the concentrations of their components becomes increasingly crucial, as the stakes are higher. It is thus reassuring to observe that subunits used in large complexes are not only highly expressed, but also less noisy than other similarly abundant proteins.

**Figure 4:**
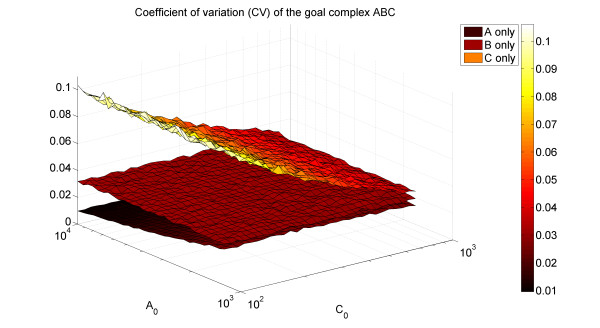
**(Color) Variation in the goal complex as a result of variation in one component (model)**. For various values of A_0 _and C_0 _in our complex formation model (B_0 _= 1000 fixed, and C_0_<B_0_<A_0_), we plot the CV of the goal complex ABC, when only one component (bottom to top manifolds: A, B, C, respectively) is allowed stochastic variation. It can be seen that the CV of the goal complex is most sensitive to variations in the concentration of C, its least abundant component.

Another property of the noise in complexes is that the noise level of the least abundant protein in a complex is lower than expected. This is also indicative of optimization. To show this, we demonstrate that in our 3-component complex formation model, variation in complex levels is most sensitive to fluctuations in the level of the least abundant protein. We look at the production of the goal complex ABC when fixing the levels of two of the components, and letting the third to fluctuate, its level being drawn from a normal distribution with standard deviation equals to the square root of its mean. Figure 4 demonstrates that the CV of the goal complex concentration is always higher when the variation is in the component of lowest abundance, thus demonstrating that variations in its levels are more crucial. This fits nicely with the empirical findings of higher regulation of the least abundant protein, compared to no regulation of the most abundant protein.

### Similarity of transcript length in a complex

The similarity in transcript lengths of complex subunits might turn useful to make complex assembly more efficient- when complex subunits have largely different lengths, and given the dependence of the transcription and translation time on length, the cell would have spent extra effort to coordinate protein synthesis to bring the complete complex together just in time (see[13] for the analysis of complex just in time synthesis vs. just in time assembly).

## Conclusion

In summary, based on several sources of experimental data, we have shown that the composition of protein complexes in yeast is optimized towards more efficient complex generation. In particular, not only that protein abundances are similar, but their response to minimal media is also similar, providing another evidence for optimization. Moreover, the variation in protein levels is optimized for a more robust complex production by minimizing the stochastic fluctuations in the concentration of large complexes and in the concentration of the least abundant protein in a complex. These results are indicative of transcriptional, as well as post-transcriptional regulation at the global level of the yeast cell. Preliminary results show that similarity in protein levels in a complex is mainly due to regulation of translational activity rather than degradation rate. However, the precise combination of regulatory mechanism that leads to the optimized levels is still to be elucidated.

For Prokaryotes, the simplest method to guarantee coexpression of genes is to couple them in a single operon. Having all genes of a given complex bundled together in a single operon with a single promoter will yield the exact amount of proteins needed for the synthesis of the complex under all circumstances. However, this simplistic approach requires the genome to contain multiple copies for each gene that appears in more than one complex. Instead, most Eukaryotes adopt a more flexible scheme, as each gene appears only once (or very few times) in the genome. However, there is a price to pay in the form of additional regulation mechanisms at various levels, which are necessary for maintaining efficient usage of the available resources. These include keeping subunits of a complex at similar levels, similar response to external change of conditions, and low noise.

Our analysis of experimental data was performed on the yeast, a unicellular organism. In more complex multicellular organisms, more constraints apply due the diversity in the function of the various cells. In particular, it would be interesting to explore the potential role that improper function of the regulation of proteins level and noise we found might have in any disease state. Future experimental advances providing data on protein complexes and protein levels in higher organism will enable study of the implementation of the aforementioned optimization principles in complex organisms, in health and in disease.

## Methods

### A model of complex formation

The model of complex formation we analyze was introduced in[19], where more technical details are given. In short, we study a complex made up of 3 different components: A, B, and C. We denote the concentrations of the three components of the complex by [A], [B], and [C], and the concentrations of the complexes they form by [AB], [AC], [BC], and [ABC]. The latter is the concentration of the complete complex that is the desired outcome of the production, while the first three describe different sub-complexes which are formed (each of which is composed of two components). A typical kinetic equation looks like:

$$\frac{d[A]}{dt}=k_{d_{A,B}}[AB]+k_{d_{A,C}}[AC]+k_{d_{A,BC}}[ABC]-k_{a_{A,B}}[A][B]-k_{a_{A,C}}[A][C]-k_{a_{A,BC}}[A][BC],$$

where $k_{a_{x,y}}$ ($k_{d_{x,y}}$) are the association (dissociation) rates of the subcomponents *x *and *y *to form the complex *xy *(direct three-body interactions, i.e., generation or decomposition of ABC out of A, B, and C, are neglected). Similar equations hold for the other 6 sub-complexes. Denoting the total number of A, B, and C particles by A_0_, B_0_, and C_0_, respectively, we may write the conservation of material equations as follows: [A] + [AB] + [AC] + [ABC] = A_0_, and similarly for the B's and the C's.

We look for the steady-state solution of these equations, where all time derivatives vanish. For simplicity, we consider the totally symmetric situation, where all the ratios of association coefficients to their corresponding dissociation coefficients are equal, i.e., the ratios $k_{d_{x,y}}$/$k_{a_{x,y}}$ are all equal to X_0_, where X_0 _is a constant with concentration units. In this case, measuring concentrations in units of X_0_, the reaction equations are all solved by the substitutions [AB] = [A] [B], [AC] = [A] [C], [BC] = [B] [C], and [ABC] = [A] [B] [C], and one needs only to solve the set of three conservation of material equations, which take the form: [A] + [A] [B] + [A] [C] + [A] [B] [C] = A_0_, and similarly for the B's and the C's. These equations allow for an exact and straightforward (albeit cumbersome) analytical solution, which we explore throughout the manuscript.

An efficiency of complex formation can be defined in our model as the ratio of the number of full complexes to the amount of the component with lowest abundance: [ABC]/min(A_0_, B_0_, C_0_). Alternatively, the efficiency could be defined in terms of the total utilization of proteins: [ABC]/(A_0_+B_0_+C_0_). Both definitions lead to the same conclusion that protein synthesis is optimal whenever all (or the two most abundant, in the case of the first definition) proteins have similar levels. The first efficiency measure is somewhat advantageous as it ignores the obvious waste of excess subunits, and thus it underscores the additional harmful effect of concentration imbalance on the reaction dynamics (see also in [19]).

### Randomization schemes

To estimate the significance of our results, we used two methods of randomization of protein properties. To demonstrate the methods, consider for example the study of protein levels. In the first method we gave each protein the level of another, uniformly chosen protein (out of proteins which participate in complexes). This preserves the distribution of protein levels, but not the total number of times a given level appears in the complexes. In the second method, which preserves the latter, but not the former, we assigned each protein the level of another protein, chosen with probability proportional to the actual number of times it appears in the complex dataset. To compute P-value, we calculated the variance in the distribution of the randomized results and assumed they are normally distributed. We verified that all results are significant in both methods, whenever the application of both is relevant.

To control for abundance, we replaced the examined property of a protein with another protein chosen among those having a concentration close to the original one, where 'close' means the absolute value of the natural logarithm of the ratio of concentrations is less than 1. To control for essentiality, we exchanged only proteins which were either both essential or both non-essential.

## Authors' contributions

SC performed the computational analysis. SC, EYL and EE conceived and designed the study. SC and EE wrote the paper. All authors read and approved the final manuscript.

## Supplementary Material

Additional file 1 **A list of the complexes**. The file contains a list of all complexes (extracted from MIPS, 2007). Each row in the file contains the systematic names of the proteins in one complex. Click here for file

Additional file 2 **Extended discussion**. The file contains complementary discussion of few specific points. • A list of complexes with uniform response to change of environmental conditions, and an analysis of their properties in view of their role in cell cycle. • A discussion of the relation between our findings to the dosage balance hypothesis. • Model results for the optimal response to transition to minimal media when all subunits are allowed to change. • Illustration that complex proteins have lower noise. • A discussion of alternative hypotheses that could explain the low noise in large complexes, the high level of the least abundant protein in a complex, and the similarity of transcript length in a complex. Click here for file

## References

[B1] Alberts B (1998). The cell as a collection of protein machines: preparing the next generation of molecular biologists. Cell.

[B2] Ito T, Tashiro K, Muta S, Ozawa R, Chiba T, Nishizawa M, Yamamoto K, Kuhara S, Sakaki Y (2000). Toward a protein-protein interaction map of the budding yeast: A comprehensive system to examine two-hybrid interactions in all possible combinations between the yeast proteins. Proceedings of the National Academy of Sciences of the United States of America.

[B3] Uetz P, Giot L, Cagney G, Mansfield TA, Judson RS, Knight JR, Lockshon D, Narayan V, Srinivasan M, Pochart P (2000). A comprehensive analysis of protein-protein interactions in Saccharomyces cerevisiae. Nature.

[B4] Gavin AC, Aloy P, Grandi P, Krause R, Boesche M, Marzioch M, Rau C, Jensen LJ, Bastuck S, Dumpelfeld B (2006). Proteome survey reveals modularity of the yeast cell machinery. Nature.

[B5] Krogan NJ, Cagney G, Yu H, Zhong G, Guo X, Ignatchenko A, Li J, Pu S, Datta N, Tikuisis AP (2006). Global landscape of protein complexes in the yeast Saccharomyces cerevisiae. Nature.

[B6] Gavin AC, Bosche M, Krause R, Grandi P, Marzioch M, Bauer A, Schultz J, Rick JM, Michon AM, Cruciat CM (2002). Functional organization of the yeast proteome by systematic analysis of protein complexes. Nature.

[B7] Ho Y, Gruhler A, Heilbut A, Bader GD, Moore L, Adams SL, Millar A, Taylor P, Bennett K, Boutilier K (2002). Systematic identification of protein complexes in Saccharomyces cerevisiae by mass spectrometry. Nature.

[B8] Barabasi AL, Oltvai ZN (2004). Network biology: understanding the cell's functional organization. Nature reviews.

[B9] Hughes TR, Marton MJ, Jones AR, Roberts CJ, Stoughton R, Armour CD, Bennett HA, Coffey E, Dai H, He YD (2000). Functional discovery via a compendium of expression profiles. Cell.

[B10] Ge H, Liu Z, Church GM, Vidal M (2001). Correlation between transcriptome and interactome mapping data from Saccharomyces cerevisiae. Nature genetics.

[B11] Grigoriev A (2001). A relationship between gene expression and protein interactions on the proteome scale: analysis of the bacteriophage T7 and the yeast Saccharomyces cerevisiae. Nucleic acids research.

[B12] Jansen R, Greenbaum D, Gerstein M (2002). Relating whole-genome expression data with protein-protein interactions. Genome research.

[B13] de Lichtenberg U, Jensen LJ, Brunak S, Bork P (2005). Dynamic complex formation during the yeast cell cycle. Science.

[B14] Papp B, Pal C, Hurst LD (2003). Dosage sensitivity and the evolution of gene families in yeast. Nature.

[B15] Huh WK, Falvo JV, Gerke LC, Carroll AS, Howson RW, Weissman JS, O'Shea EK (2003). Global analysis of protein localization in budding yeast. Nature.

[B16] Dezso Z, Oltvai ZN, Barabasi AL (2003). Bioinformatics analysis of experimentally determined protein complexes in the yeast Saccharomyces cerevisiae. Genome research.

[B17] Giaever G, Chu AM, Ni L, Connelly C, Riles L, Veronneau S, Dow S, Lucau-Danila A, Anderson K, Andre B (2002). Functional profiling of the Saccharomyces cerevisiae genome. Nature.

[B18] Sopko R, Huang D, Preston N, Chua G, Papp B, Kafadar K, Snyder M, Oliver SG, Cyert M, Hughes TR (2006). Mapping pathways and phenotypes by systematic gene overexpression. Molecular cell.

[B19] Carmi S, Levanon EY, Havlin S, Eisenberg E (2006). Connectivity and expression in protein networks: proteins in a complex are uniformly expressed. Physical review.

[B20] Mewes HW, Frishman D, Guldener U, Mannhaupt G, Mayer K, Mokrejs M, Morgenstern B, Munsterkotter M, Rudd S, Weil B (2002). MIPS: a database for genomes and protein sequences. Nucleic acids research.

[B21] Ghaemmaghami S, Huh WK, Bower K, Howson RW, Belle A, Dephoure N, O'Shea EK, Weissman JS (2003). Global analysis of protein expression in yeast. Nature.

[B22] Birchler JA, Riddle NC, Auger DL, Veitia RA (2005). Dosage balance in gene regulation: biological implications. Trends Genet.

[B23] Veitia RA (2004). Gene dosage balance in cellular pathways: implications for dominance and gene duplicability. Genetics.

[B24] Newman JR, Ghaemmaghami S, Ihmels J, Breslow DK, Noble M, DeRisi JL, Weissman JS (2006). Single-cell proteomic analysis of S. cerevisiae reveals the architecture of biological noise. Nature.

[B25] Ito T, Chiba T, Ozawa R, Yoshida M, Hattori M, Sakaki Y (2001). A comprehensive two-hybrid analysis to explore the yeast protein interactome. Proceedings of the National Academy of Sciences of the United States of America.

[B26] Belle A, Tanay A, Bitincka L, Shamir R, O'Shea EK (2006). Quantification of protein half-lives in the budding yeast proteome. Proceedings of the National Academy of Sciences of the United States of America.

[B27] Brockmann R, Beyer A, Heinisch JJ, Wilhelm T (2007). Posttranscriptional expression regulation: what determines translation rates?. PLoS computational biology.

[B28] Mewes HW, Frishman D, Mayer KF, Munsterkotter M, Noubibou O, Pagel P, Rattei T, Oesterheld M, Ruepp A, Stumpflen V (2006). MIPS: analysis and annotation of proteins from whole genomes in 2005. Nucleic acids research.

[B29] Hunte C, Koepke J, Lange C, Rossmanith T, Michel H (2000). Structure at 2.3 A resolution of the cytochrome bc(1) complex from the yeast Saccharomyces cerevisiae co-crystallized with an antibody Fv fragment. Structure.

[B30] Fraser HB, Hirsh AE, Giaever G, Kumm J, Eisen MB (2004). Noise minimization in eukaryotic gene expression. PLoS biology.

[B31] Miura F, Kawaguchi N, Sese J, Toyoda A, Hattori M, Morishita S, Ito T (2006). A large-scale full-length cDNA analysis to explore the budding yeast transcriptome. Proceedings of the National Academy of Sciences of the United States of America.

[B32] Eisenberg E, Levanon EY (2003). Human housekeeping genes are compact. Trends Genet.

[B33] Warringer J, Blomberg A (2006). Evolutionary constraints on yeast protein size. BMC evolutionary biology.

[B34] Hartwell LH, Hopfield JJ, Leibler S, Murray AW (1999). From molecular to modular cell biology. Nature.

