# Supplementary material for: Efficiency of complex production in changing environment
Source: BMC Syst Biol. 2009 Jan 7;3:3. doi: 10.1186/1752-0509-3-3 (PMC2647903; doi:10.1186/1752-0509-3-3)
Supplement: Additional file 2 — Extended discussion. The file contains complementary discussion of few specific points. • A list of complexes with uniform response to change of environmental conditions, and an analysis of their properties in view of their role in cell cycle. • A discussion of the relation between our findings to the dosage balance hypothesis. • Model results for the optimal response to transition to minimal media when all subunits are allowed to change. • Illustration that complex proteins have lower noise. • A discussion of alternative hypotheses that could explain the low noise in large complexes, the high level of the least abundant protein in a complex, and the similarity of transcript length in a complex. [file 1752-0509-3-3-S2.pdf]

Supplementary material for the article:

Efficiency of complex production in changing environment

S. Carmi, E.Y. Levanon, and E. Eisenberg

Uniform complexes

A list of complexes in which all proteins change their levels in a similar fashion when growth conditions are changed from YEPD to SD is given below (Table 1).

Class	MIPS no.	Proteins (systematic name)	Description from MIPS or GO term[a]
Minimal	420.30	Q0105 YBL045C YDR529C YEL024W YFR033C YGR183C YHR001W-A YJL166W YOR065W YPR191W	Cytochrome bc1 complex (Ubiquinol-cytochrome c reductase complex, complex III)
Constant	550.1.77	Q0080 YAL002W YDL077C YDL087C YDR080W YGR013W YLR148W YLR396C YMR231W YPL045W	Probably membrane biogenesis and traffic
	500.60.20	Q0140 YBL090W YBR146W YBR251W YDL045W-A YDR041W YDR175C YDR337W YDR347W YER050C YFR049W YGL129C YGR084C YGR165W YGR215W YHL004W YHR075C YIL093C YJR101W YJR113C YKL003C YKL155C YMR158W YMR188C YNL137C YNL306W YNR037C YOR158W YPL013C YPL118W YPR166C	Translation complexes: mitochondrial ribosomal small subunit
	550.2.48	YAL016W YDL134C YDL188C YDR188W YDR212W YGL190C YHR033W YIL142W YJL014W YJR064W YNL154C	Cytoskeleton organization and biogenesis
	140.20.30	YAL029C YBR172C YHR023W YKL079W YKL129C YMR109W YOR326W	Cytoskeleton: Actin-associated motorproteins
	550.1.5	YAL041W YBR200W YER114C YLR357W	Probably cell cycle
	410.30	YBL023C YBR060C YBR202W YEL032W YGL113W YGL201C YHR118C YJL090C YJL194W YLL004W YLR103C YLR274W YML065W YNL261W YPR019W YPR162C	Replication complexes: Pre-replication complex (pre-RC)
	410.35	YBL023C YBR060C YBR202W YBR278W YDL102W YEL032W YGL201C YHR118C YJL090C YJR006W YJR043C YLL004W YLR103C YLR274W	Replication complexes: Replication complex

		YML065W YNL261W YNL262W YPR019W YPR162C YPR175W	
	550.1.169	YBL046W YDL112W YDR075W YDR379W YER155C YGR192C YML010W YNL201C	Probably signaling
	550.2.234	YBL046W YDR075W YDR212W YHR011W YHR033W YHR084W YIL142W YJL014W YNL201C	Protein folding
	510.40.20	YBL093C YBR193C YBR253W YDL005C YDR308C YER022W YER179W YGL025C YGL151W YGR104C YHR041C YHR058C YLR071C YMR112C YNL236W YNR010W YOL051W YOL135C YOR174W YPL129W YPR168W	Transcription complexes/Transcriptosome: Kornberg's mediator (SRB) complex
	550.2.112	YBR081C YCL010C YDR448W YGL112C YGR252W YHR099W YMR223W YMR319C YPL254W	Chromatin
	410.40.30	YBR087W YJR068W YNL290W YOL094C YOR217W	Replication complexes: Replication factor C complex
	390	YBR221C YER178W YFL018C YGR193C YNL071W	Pyruvate dehydrogenase
	270.20.30	YBR233W-A YDR016C YDR201W YDR320C-A YGL061C YGR113W YKL052C YKR037C YKR083C	Kinetochore protein complexes: Dam1 protein complex
	510.190.50	YBR289W YDR073W YHL025W YJL176C YMR033W YNR023W YOR290C YPL016W YPL129W YPR034W	Transcription complexes/Transcriptosome: SWI/SNF transcription activator complex
	550.2.214	YCR077C YDL065C YDL160C YER112W YJL124C YNL118C	Deadenylation-dependent decapping of nuclear-transcribed mRNA
	550.2.215	YCR077C YDL065C YDL160C YER112W YGL121C YJL124C YNL118C YNL147W	Deadenylation-dependent decapping of nuclear-transcribed mRNA
	550.2.203	YDL047W YFR021W YFR040W YGL190C YLR222C YNL242W YPL258C	Type 2A-related serine-threonine phosphatase that functions in the G1/S transition of the mitotic cycle; cytoplasmic and nuclear protein that modulates functions mediated by Pkc1p including cell wall and actin cytoskeleton organization
	550.2.368	YDR032C YDR422C YGL115W YOR018W YOR267C YPR160W	
	550.2.190	YDR092W YGL087C YOL081W	Protein polyubiquitination,

		YOR220W	postreplication repair
	550.2.329	YDR388W YHL030W YIL156W YJR139C YLL039C YML123C YPL249C	Protein deubiquitination
	550.1.150	YEL015W YML091C YNL118C YOL149W	Probably RNA metabolism
	260.20.40	YER157W YGL005C YGL223C YGR120C YML071C YNL041C YNL051W YPR105C	Intracellular transport complexes: Golgi transport complex
	550.1.226	YGR180C YIL070C YJL026W YJL115W YJR140C YPL153C	Probably transcription/DNA maintanance/chromatin structure
	260.80	YLR148W YLR396C YMR231W YPL045W	Class C Vps protein complex
Rich	550.1.116	YAL035W YDR101C YER036C YPR036W	Probably protein synthesis turnover
	550.2.104	YAL036C YHR027C YJL138C YMR012W	Translational initiation
	550.2.474	YBL002W YGR067C YJL080C YMR012W YNL085W	
	360.10.10	YBL041W YBR173C YER012W YER094C YFR050C YGL011C YGR135W YGR253C YJL001W YML092C YMR314W YOL038W YOR157C YOR362C YPR103W	Proteasome: 20S proteasome
	550.3.38	YBR031W YDR101C YJL122W YLL045C YOL041C YOR312C	Ribosomal export complex
	550.3.51	YBR079C YDR091C YDR429C YER025W YLR192C YMR146C YMR309C YOR361C YPR041W	Eukaryotic translation initiation factor 3 complex
	520.10.20	YBR171W YLR292C YOR254C YPL094C	Translocon: Sec62-63 complex (A protein complex involved in the posttranslational targeting of proteins to the ER)
	550.1.53	YBR207W YEL022W YER021W YER110C YFL041W YLR342W YOL086C	Probably intermediate and energy metabolism
	550.3.87	YCR057C YDR449C YJL069C YLR129W YLR222C YLR409C	Pwp2p-containing subcomplex of 90S preribosome
	550.2.108	YCR084C YDR211W YGR083C YKR026C YLR291C YOR260W	Regulation of translational initiation
	550.3.15	YDL014W YDR117C YGL078C YGL173C YGR159C YGR162W YJL033W YOL076W YOR206W YOR310C	Small subunit processome complex
	260.30.10	YDL145C YDL192W YDR238C YFR051C YGL137W YIL076W YNL287W YPL010W	Intracellular transport complexes: COPI (COPI vesicle coatomer complex)

	500.10.30	YDR211W YGR083C YKR026C YLR291C YOR260W	Translation initiation factor
	550.3.52	YDR211W YER025W YFL039C YGR083C YGR159C YLR291C YOR260W YOR361C YPL237W	Translation initiation factor
	550.2.158	YDR212W YDR342C YGL195W YHR199C YJR132W YLR326W YLR347C YNL323W	
	550.3.17	YER165W YGR159C YGR162W YJL033W YJL050W YLR002C YMR229C YOR206W YOR310C YPL263C	RNA-processing complex
	550.3.4	YER177W YGL078C YGR159C YLR410W YOR310C	Ribosomal biogenesis
	550.2.171	YGL141W YGR103W YKL021C YMR049C YNL061W YPR016C	
	550.3.40	YLR196W YLR410W YOL041C YOL077C	rRNA processing

[a] Whenever the description of the complex was given in the MIPS data set, it is reported. Otherwise, we report the GO term with lowest P-value common to all genes in the complex (www.yeastgenome.org).

Table 1- A list of 46 complexes whose protein levels change uniformly, along with the MIPS identifier, protein systematic names, and complex function (when available).

Properties of uniform complexes

Our results for the uniform complexes are complementary to the results of ref[1], where it was shown that cell-cycle complexes are made up from both constitutively expressed (static) and periodically expressed (dynamic) proteins. We show that for a steady-state growth in different environments (as opposed to periodic changes in the course of cell cycle), the levels of all (or most) subunits tend to change in the same direction. It is interesting to compare the uniform complexes with the cell cycle information of ref[1], which provides a classification of proteins into dynamic and static, and the precise position in the cell cycle in which the dynamic proteins are expressed. We integrated that information with our 46 uniform complexes. In Figure S1, we show the fraction of dynamic proteins within each complex. It can be seen that only 12/46 uniform complexes contain any dynamic protein (compared to 22±3, if each protein in a complex is dynamic with probability 0.086, which is the fraction of complex proteins which are dynamic).

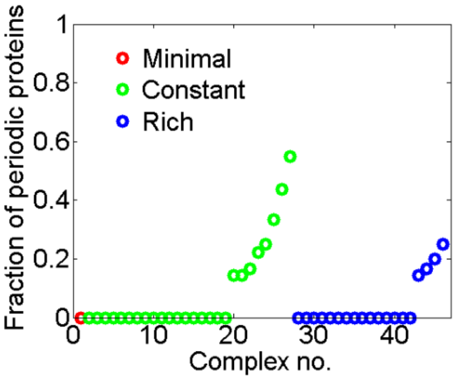

Figure S1- For each of the uniform complexes, we plot the fraction of proteins which are expressed periodically[1]. The complexes in each group (minimal (red), constant (green), and rich (blue)), were ordered by their fraction of periodic proteins.

In Figure S2, we plot the position within the cell cycle in which each dynamic protein is expressed (zero is M/G_1 boundary). The two complexes which are highly dynamic and the expressions of their proteins are most concerted (no. 26 and 27 below) are the pre-replication, and replication complex, respectively.

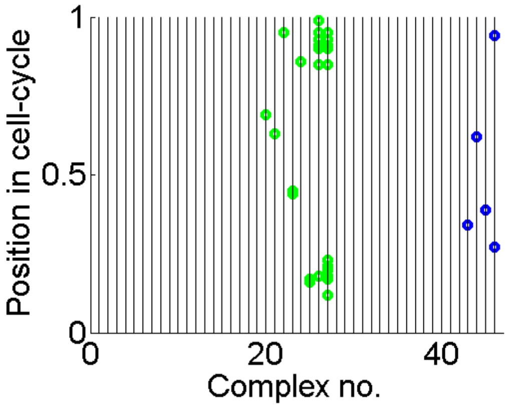

Figure S2- For each of the uniform complexes, we plot the position within the cell cycle of all of its periodic proteins[1]. The complexes are ordered as in Figure S1.

Focusing attention to the function of the uniform complexes, we could identify 12/19 of the rich state complexes as related to translation or ribosome biosynthesis, in accordance with previous studies[2-4] which showed that the yeast is very sensitive to deviations from uniformity in ribosomal proteins.
The regulation of the change in protein levels is probably harder to achieve as the complex gets larger. Indeed, the average number of subunits in uniform complexes is smaller than in non-uniform complexes (average no. of subunits per complex 8.3 vs. 12.3, P=0.02, t-test).

The dosage balance hypothesis

The dosage balance hypothesis[3] states that "an imbalance in the concentration of the subcomponents of a protein-protein complex can be deleterious". Thus, "underexpression and overexpression of protein complex subunits should lower fitness, and … the accuracy of transcriptional co-regulation of subunits should reflect the deleterious consequences of imbalance." Our results that protein levels in a complex are uniform in different growth conditions demonstrate that regulation of subunit levels exists even at the protein level. This regulation, we argued, is aimed to optimize complex production. However, a natural question is whether these results can be solely explained based on a potentially harmful effect of incomplete subcomplex, or is there further regulation towards minimization of the waste of resources. Here we show that the dosage balance is not likely to be attributed solely to the harmful effects of partial complexes as it does not correlate with haplo-insufficiency and over-expression toxicity.

First, we used a data set which divides all proteins into haplo-sufficient and haplo-insufficient based on the growth pattern after a deletion of one allele of their gene[2]. We call each complex with at least one haplo-insufficient protein a haplo-insufficient complex. If harmful effects of complex subunits were to explain our findings, one would have expected the protein levels[5] of the haplo-insufficient complexes to be more tightly regulated, and thus more uniform. However, in reality the converse it true - the variance in the (log of the) levels of the (245) haplo-insufficient complexes is slightly (but not significantly) higher than the levels of the (297) haplo-sufficient complexes (P=0.08).

Second, we studied the list of genes that are toxic when over-expressed[6]. As before, we designate as toxic complexes these complexes which contain at least one toxic over-expressed gene. Here too, the variance in the (log of the) levels of the (381) toxic complexes is higher than the (161) non-toxic ones (P=0.003).

Finally, the harmful effects of dosage imbalance are also unlikely to explain the maintenance of protein levels across different conditions. To show that, we examine the fraction of haplo-insufficient and toxic complexes that show uniform direction of change between minimal and rich conditions. We find that the fraction of complexes with uniform direction of change is lower in haplo-insufficient and toxic complexes (considering only complexes with at least four subunits). While only 9.2% of the haplo-insufficient complexes exhibit uniform change, 12.5% of the haplo-sufficient complexes are uniform. Similarly, only 8.4% of the toxic complexes are uniform, compared to 17.9% of the non-toxic complexes. Thus, complexes that consist of proteins with harmful effect of dosage imbalance do not show more uniform levels across different condition than other complexes.

In conclusion, the uniformity in the protein levels in a complex cannot be explained solely by the tight regulation of those proteins which are highly sensitive to change in their levels. Instead, our results suggest dosage optimization across the board, in accordance with the economic usage principle.

Model results for change in all components

Figure 3 in the manuscript reports results for the number of saved molecules following a change in one component out of three (A,B,C) to lower the concentration of the target complex ABC. It can be seen from the figure that changes in more abundant components results in more saved molecules. This conclusion is valid even if we allow all three components to change. To show this, we fix the initial values of (A_0,B_0,C_0) and calculate the level of ABC from the model. We then fix the new ABC level to be 10% of the original ABC, and allow of A_0 and B_0 to change. We scan all possible values of A_0 and B_0 (C_0 is automatically determined by the restriction on the final level of ABC), and select the optimal set of values which yields the largest decrease in the total number of molecules used relative to the number originally used (i.e., A_0+B_0+C_0). We then repeated the analysis for several initial values of (A_0,B_0,C_0), and plotted the ratio between the new and original concentration (in the optimal configuration), for A_0, B_0, and C_0. It can be seen that the relative concentration of A_0 (the most abundant component) compared to the original concentration is the smallest. The relative concentration of B_0 is larger, and for C_0 (the least abundant component) is the largest. Therefore, in the optimal case, the more abundant component decreases more.

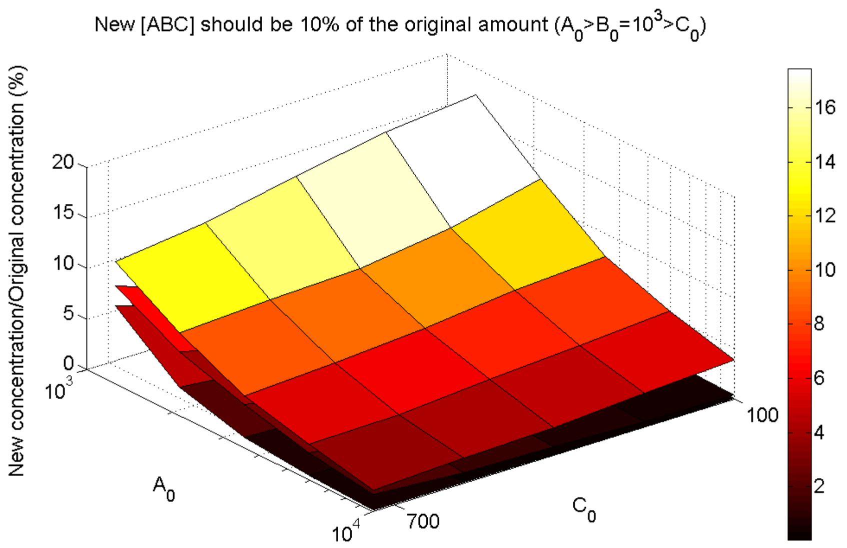

Figure S3. The ratio (percent) between the new and old concentration of A,B, and C (bottom to top surfaces, respectively). The x and y-axes denote the initial concentration A_0 and C_0. All three components are allowed to change, constrained by the final concentration of the ABC complex being %10 of the original, and the optimal solution (in terms of minimum total number of molecules) is chosen.

Lower noise of complex proteins

In Figure S4 below, we plot the distribution of CV (noise) values for complex and non-complex proteins. It can be seen that complex proteins exhibit lower values of noise ($P<10^{-14}$).

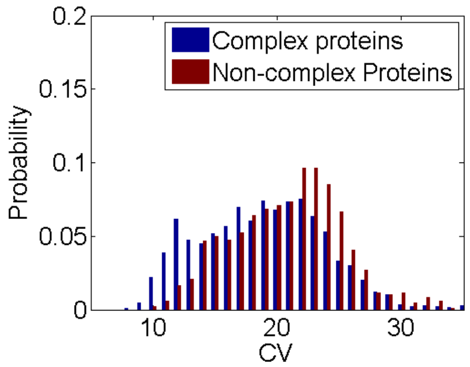

Figure S4. Distribution of CV (noise) values, for complex proteins and non-complex proteins[7].

An alternative explanation to the low noise in large complexes

Large complexes contain higher fraction of essential proteins[8] (7% more). This could provide an alternative explanation to the reduced noise in large complexes, as essential proteins are less noisy[7]. To check whether this effect might account for the lower noise, we compared the components of large complexes to random proteins, but controlled for essentiality by keeping the total number of essential proteins constant. This resulted in an average level of noise (CV) of 18.52 compared to 19.0±0.12 expected by chance ($P<10^{-4}$; the lower noise in large complexes remains significant even when abundance is controlled for ($P\approx0.003$)). Thus we conclude that the low noise in large complexes cannot be explained solely by the higher fraction of essential or abundant proteins.

Note that even the usage of more essential proteins in large complexes does, by itself, increase efficiency. Here we observe an additional effect, which might be explained as a supplementary optimization towards more economic usage of resources.

An alternative explanation to the high level of the least abundant protein in a complex

The least abundant protein of each complex was shown to have higher concentration and, consequently, lower noise. We interpreted this observation in terms of a kinetic argument showing that the variation in the level of the goal complex is most sensitive to the concentration of the least abundant protein. If this explanation holds, it is yet another manifestation of efficiency.

However, the higher concentration of the least abundant protein might be explained as a consequence of complexes having more uniform subunit abundances than random: if the variance of intra-complex levels is low, then the lowest concentration subunit would always have higher average abundance than if the variance of intra-complex abundance were high.

To test this alternative explanation, we look at the concentration of the most abundant protein in a complex. If the alternative explanation is true, one expects the concentration of the protein of highest level to be lower than expected by chance. We find that the average concentration of the least abundant protein is 1310 (molecules/cell) compared to 960 after randomization, a 36% increase, while the concentration of the most abundant protein is 145,000, compared to 157,000 after randomization, which is a decrease of only 8%. Thus we observe that the protein of lowest abundance exhibits a much stronger increase in its level, indicating another level of regulation beyond the tendency towards overall low variance, which attests for the validity of our kinetic argument.

An alternative explanation to the similarity of transcript length in a complex

It has been previously reported[9] and can be easily verified that biochemical activity is correlated with protein length. Thus, the similarity of transcript length in a complex could be alternatively explained due to the common function of complex members. To refute this alternative hypothesis, we performed the following computation. We downloaded GO annotation for the yeast genome from SGD (*www.yeastgenome.org*). For each gene, we extracted all "molecular function" GO terms which are associated with it (where we considered terms at the third level of the tree, altogether adding up to 79 terms). Without controls, to establish the fact that complex subunits have similar transcript length, we calculated the variance of the (log of the) transcript lengths and averaged over all complexes. We then repeated the same calculation when the complex proteins were shuffled. However, when shuffling the complex proteins, we only allowed an exchange of two proteins which share at least one GO molecular function term. Thus, we control for the effect of the similar functions of the complex subunits (this, in addition to control for abundance, since it is also known that gene length is related to abundance). While the real variance was 0.27, the variance after randomization was 0.316+-0.012, yielding P-value of about 10^{-5}. Without any controls the variance after randomization is higher (0.334+-0.014, $P \approx 2 \cdot 10^{-7}$), leading to stronger result. However, we can still conclude that the tendency of

complex subunits to contain genes with similar transcript size is not in all due to the similar function of the complex subunits.

1. de Lichtenberg U, Jensen LJ, Brunak S, Bork P: **Dynamic complex formation during the yeast cell cycle**. *Science (New York, NY* 2005, **307**(5710):724-727.
2. Deutschbauer AM, Jaramillo DF, Proctor M, Kumm J, Hillenmeyer ME, Davis RW, Nislow C, Giaever G: **Mechanisms of haploinsufficiency revealed by genome-wide profiling in yeast**. *Genetics* 2005, **169**(4):1915-1925.
3. Papp B, Pal C, Hurst LD: **Dosage sensitivity and the evolution of gene families in yeast**. *Nature* 2003, **424**(6945):194-197.
4. Warner JR: **The economics of ribosome biosynthesis in yeast**. *Trends in biochemical sciences* 1999, **24**(11):437-440.
5. Ghaemmaghami S, Huh WK, Bower K, Howson RW, Belle A, Dephoure N, O'Shea EK, Weissman JS: **Global analysis of protein expression in yeast**. *Nature* 2003, **425**(6959):737-741.
6. Sopko R, Huang D, Preston N, Chua G, Papp B, Kafadar K, Snyder M, Oliver SG, Cyert M, Hughes TR *et al*: **Mapping pathways and phenotypes by systematic gene overexpression**. *Molecular cell* 2006, **21**(3):319-330.
7. Newman JR, Ghaemmaghami S, Ihmels J, Breslow DK, Noble M, DeRisi JL, Weissman JS: **Single-cell proteomic analysis of S. cerevisiae reveals the architecture of biological noise**. *Nature* 2006, **441**(7095):840-846.
8. Giaever G, Chu AM, Ni L, Connelly C, Riles L, Veronneau S, Dow S, Lucau-Danila A, Anderson K, Andre B *et al*: **Functional profiling of the Saccharomyces cerevisiae genome**. *Nature* 2002, **418**(6896):387-391.
9. Warringer J, Blomberg A: **Evolutionary constraints on yeast protein size**. *BMC evolutionary biology* 2006, **6**:61.
